# Mid-arm muscle circumference as a surrogate in predicting insulin resistance in non-obese elderly individuals

**DOI:** 10.18632/oncotarget.19340

**Published:** 2017-07-18

**Authors:** Yuan-Ping Chao, Yi-Fen Lai, Tung-Wei Kao, Tao-Chun Peng, Yuan-Yung Lin, Mu-Tsun Shih, Wei-Liang Chen, Li-Wei Wu

**Affiliations:** ^1^ Division of Family Medicine, Department of Family and Community Medicine, Tri-Service General Hospital and School of Medicine, National Defense Medical Center, Taipei, Taiwan, Republic of China; ^2^ Division of Geriatric Medicine, Department of Family and Community Medicine, Tri-Service General Hospital and School of Medicine, National Defense Medical Center, Taipei, Taiwan, Republic of China; ^3^ Department of Otolaryngology–Head and Neck Surgery, Tri-Service General Hospital and School of Medicine, National Defense Medical Center, Taipei, Taiwan, Republic of China; ^4^ Graduate Institute of Medical Sciences, National Defense Medical Center, Taipei, Taiwan, Republic of China; ^5^ Division of Urology, Department of Surgery, Tri-Service General Hospital and School of Medicine, National Defense Medical Center, Taipei, Taiwan, Republic of China

**Keywords:** anthropometric parameters, insulin resistance, elderly, NHANES

## Abstract

The homeostatic model assessment of insulin resistance (HOMA-IR) was used to measure the degree of insulin resistance (IR). Previous literature revealed that mid-arm muscle circumference (MAMC) is one of the anthropometric indicators for nutritional status and the relationship between MAMC and HOMA-IR remains uncertain in the obese and non-obese elderly individuals. The present study included 5,607 participants aged between 60 to 84 years old, using data from the 1999 to 2006 National Health and Nutrition Examination Survey (NHANES). To further explore the association between HOMA-IR and MAMC in the obese and non-obese elderly population using multivariate Cox regression analyses, we divided the participants into obese (BMI ≥ 30 kg/m^2^) group and non-obese (19 ≤ BMI < 30 kg/m^2^) group in this study; each group was then divided into quartiles based on their MAMC levels. A positive association was noted between the MAMC and HOMA-IR in all of the designed models initially. After adjusting for multiple covariates, a higher level of the MAMC was significantly associated with elevated HOMA-IR (*P* < 0.05) in the non-obesity group, which was not the case in the obesity group. Additionally, subjects in the higher quartiles of MAMC tended to have higher HOMA-IR with a significant association (*P* for trend = 0.003 in model 1; *P* for trend < 0.001 in model 2, 3, and 4). These results demonstrated that the MAMC can be an auxiliary indicator of HOMA-IR in non-obese elderly individuals and may have substantial additional value in screening for IR if well extrapolated.

## INTRODUCTION

Insulin is characterized by its ability to increase the rate of blood sugar metabolism in peripheral tissues. Insulin resistance (IR) is the condition in which peripheral tissues are unable to properly extract glucose from the blood. Therefore, the insulin level increases in the circulation when IR develops [1]. Matthews *et al.* first described the homeostatic model assessment of insulin resistance (HOMA-IR) in 1985 [2]. HOMA-IR has been used to represent IR in epidemiological studies due to its convenience, requiring evaluation of only a single plasma sample for insulin and glucose [3]. HOMA-IR is associated with cardiometabolic risk factors (CMRFs) [4] and is a single factor that may indicate angiographic coronary artery disease in non-diabetic, non-obese individuals [5]. Alptekin *et al.* suggested that first trimester screening for gestational diabetic mellitus (GDM) can be achieved based on maternal anthropometric measurements and HOMA-IR [6]. A Japanese investigation demonstrated that increased HOMA-IR independently predicted the subsequent development of hyperuricemia [7]. Moreover, sarcopenia and obesity were demonstrated to have an association with HOMA-IR in a Korean study [8].

Loss of skeletal muscle mass may impede insulin-mediated glucose disposal and exacerbate obesity-related IR [9, 10]. Lean body mass can be estimated by equations based on the mid-arm muscle circumference (MAMC) and hand grip strength (HGS) [11], and the MAMC can represent muscle mass because of its association with dual-energy X-ray absorptiometry (DEXA) [12]. According to Atkins *et al.*, the MAMC was initially positively correlated with HOMA-IR. After adjusting for body mass index (BMI), the direction of the study results changed. Low MAMC had an inverse correlation with IR [13]. Atkins *et al.* asserted that the MAMC and HOMA-IR both had a strong correlation with BMI, which may interfere with the results. To further evaluate the association between the MAMC and HOMA-IR, we divided the participants into obese and non-obese groups. We studied elderly people aged from 60 to 84 years in this study, for they were more prone to sarcopenia. The present study used the 1999–2006 National Health and Nutrition Examination Survey (NHANES) databases.

## RESULTS

### Preliminary analysis

The present study included 5,607 participants, between 60 to 84 years old with available anthropometric parameters and standardized blood samples. The relationships of the anthropometric parameters (waist circumference (WC), maximal calf circumference (MCC), thigh circumference (TC), triceps skinfold (TS), subscapular skinfold (SS) and MAMC) and HOMA-IR are presented in Table 1 with four regression models. HOMA-IR has a significant positive association with the WC and MAMC (*P* < 0.05); meanwhile, HOMA-IR has a significant negative association with the MCC (*P* < 0.05) in all designed models. Before adjustment, the β coefficient of the HOMA-IR for the MAMC was 0.267 (95% confidence intervals (CI), 0.123–0.410, *P* < 0.001); for the WC, it was 0.078 (95% CI, 0.043–0.114, *P* < 0.001); and for the MCC, it was -0.455 (95% CI, −0.590–(−)0.321, *P* < 0.001).

**Table 1:** Association between the HOMA-IR and the anthropometric parameters

Anthropometric Parameters	Model^a^ 1			Model^a^ 2			Model^a^ 3			Model^a^ 4		
	β^b^ (95% CI)		*P* Value	β^b^ (95% CI)		*P* Value	β^b^ (95% CI)		*P* Value	β^b^ (95% CI)		*P* Value
	Min	max		min	max		min	max		min	max	
**WC (cm)**	0.078		< 0.001	0.080		< 0.001	0.042		0.015	0.044		0.012
	0.043	0.114		0.043	0.118		0.008	0.076		0.01	0.078	
**MCC (cm)**	−0.455		< 0.001	−0.438		< 0.001	−0.395		< 0.001	−0.387		< 0.001
	−0.590	−0.321		−0.573	−0.303		−0.518	−0.273		−0.51	−0.264	
**TC (cm)**	0.069		0.175	0.072		0.166	0.172		< 0.001	0.171		< 0.001
	−0.031	0.170		−0.03	0.175		0.078	0.265		0.078	0.264	
**TS (mm)**	0.089		0.004	0.035		0.32	0.034		0.294	0.031		0.339
	0.029	0.148		−0.034	0.105		−0.029	0.096		−0.032	0.093	
**SS (mm)**	0.095		< 0.001	0.113		< 0.001	0.038		0.132	0.038		0.127
	0.042	0.149		0.059	0.167		−0.011	0.087		−0.011	0.088	
**MAMC (cm)**	0.267		< 0.001	0.326		< 0.001	0.226		0.001	0.213		0.002
	0.123	0.410		0.178	0.475		0.091	0.36		0.078	0.348	

^a^Adjusted covariates:

Model 1 = Unadjusted.

Model 2 = Model 1 + age, gender, race.

Model 3 = Model 2 + serum fasting glucose, serum total cholesterol, serum total bilirubin, serum albumin.

Model 4 = Model 3 + coronary heart disease, angina/angina pectoris, heart attack, stroke, cancer or malignancy.

^b^β coefficients was interpreted as change of the HOMA-IR for each increase in different anthropometric parameters.

Abbreviation: WC, waist circumference; MCC, Maximal Calf Circumference; TC, thigh circumference; TS, Triceps Skinfold; SS, Subscapular Skinfold;

MAMC, MAC (cm) - 3.14* TSF (cm).

### Study sample characteristics

Table 2 depicts the participant’s characteristics in the obesity and non-obesity groups. Among all participants, 50.4% were men. The average age was 70.30 ± 7.10 years old. Participants in the obesity group had a higher HOMA-IR value, BMI level, systolic blood pressure (SBP), glucose, alanine aminotransferase (ALT), uric acid (UA), and C-reactive protein (CRP) compared with the non-obesity group. A higher total cholesterol, total bilirubin, and albumin level were found in the non-obesity group. A lower prevalence of coronary heart disease, angina/angina pectoris, heart attack, stroke, cancer or malignancy, and smoking was noted in the obesity group. The MAMC in qualified subjects is divided into four quartiles, and the clinical characteristics of the participants in the survey are presented in Table 3. A higher HOMA-IR value, BMI level, glucose, total bilirubin, albumin, and UA, and a higher prevalence of coronary heart disease and heart attack were observed in the highest MAMC quartile compared to the lowest quartile.

**Table 2:** Characteristics of non-obese and obese participants

Characteristics of Study Participants	Non-obesity	Obesity	Total	*P* value
**Continuous variables**^a^				
**HOMA-IR, mean (SD)**	3.34 (6.45)	6.38 (8.17)	4.38 (7.23)	**< 0.001**
**Age, mean (SD)**	71.09 (7.24)	68.73 (6.52)	70.30 (7.10)	**< 0.001**
**BMI (kg/m**^2^**), mean (SD)**	25.40 (2.99)	34.78 (4.41)	28.53 (5.66)	**< 0.001**
**Systolic blood pressure (mmHg), mean (SD)**	140.71 (24.12)	140.96 (21.95)	140.80 (23.42)	**0.731**
**Glucose (mmol/L), mean (SD)**	5.85 (2.26)	6.37 (2.52)	6.02 (2.36)	**< 0.001**
**Cholesterol, total (mmol/L), mean (SD)**	5.35 (1.11)	5.29 (1.09)	5.33 (1.10)	**0.09**
**Bilirubin, total (umol/L), mean (SD)**	12.38 (6.21)	11.61 (4.55)	12.12 (5.72)	**< 0.001**
**Albumin (g/dL), mean (SD)**	4.24 (0.31)	4.15 (0.31)	4.21 (0.32)	**< 0.001**
**ALT (U/L), mean (SD)**	22.93 (35.79)	23.68 (11.76)	23.18 (29.96)	**0.391**
**Uric acid (mg/dL), mean (SD)**	5.48 (1.42)	6.08 (1.49)	5.68 (1.47)	**< 0.001**
**C-reactive protein(mg/dL), mean (SD)**	0.49 (1.17)	0.65 (0.85)	0.55 (1.07)	**<0.001**
**Categorical variables**^b^				
**Male, *n* (%)**	2001 (53.6)	823 (43.9)	2824 (50.4)	**< 0.001**
**Non-Hispanic white, *n* (%)**	2183 (58.5)	967 (51.6)	3150 (56.2)	**< 0.001**
**Coronary heart disease, *n* (%)**	364 (9.7)	206 (11.0)	570 (10.2)	**0.103**
**Angina/angina pectoris, *n* (%)**	276 (7.4)	169 (9.0)	445 (7.9)	**0.078**
**Heart attack, *n* (%)**	359 (9.6)	203 (10.8)	562 (10.0)	**0.202**
**Stroke, *n* (%)**	251 (6.7)	162 (8.6)	413 (7.4)	**0.032**
**Malignancy, *n* (%)**	709 (19.0)	286 (15.3)	995 (17.7)	**0.002**
**Smoking, *n* (%)**	507 (24.2)	163 (16.5)	670 (21.7)	**< 0.001**

Abbreviation: BMI, body mass index; SBP, systolic blood pressure; ALT, alanine aminotransferase.

^a^Values were expressed as mean (standard deviation).

^b^Values in the categorical variables were expressed as number (%).

**Table 3:** Characteristics of the study participants by mid-arm muscle circumference quartiles

	Quartiles of mid-arm muscle circumference (cm)					
Characteristics of Study Participants	Q1 (15 < 23.4 cm)	Q2 (23.4 < 25.9 cm)	Q3 (25.9 < 28.4 cm)	Q4 (28.4 ≤ 43.8 cm)	Total	*P* value
**Continuous variables**^a^						
**HOMA-IR, mean (SD)**	2.76 (5.07)	3.55 (6.39)	4.93 (9.45)	5.76 (8.81)	4.28 (7.76)	**< 0.001**
**Age, mean (SD)**	71.60 (7.41)	71.67 (7.38)	70.49 (6.81)	68.05 (6.20)	70.45 (7.12)	**< 0.001**
**BMI (kg/m**^2^**), mean (SD)**	24.24 (3.74)	27.09 (3.99)	28.57 (4.18)	31.94 (5.18)	27.97 (5.13)	**< 0.001**
**Systolic blood pressure (mmHg), mean (SD)**	143.99 (25.57)	142.37 (23.96)	139.94 (23.12)	137.15 (20.56)	140.85 (23.49)	**< 0.001**
**Glucose (mmol/L), mean (SD)**	5.67 (2.29)	5.88 (2.14)	6.17 (2.49)	6.25 (2.47)	5.99 (2.36)	**< 0.001**
**Cholesterol, total (mmol/L), mean (SD)**	5.55 (1.13)	5.38 (1.14)	5.20 (1.04)	5.17 (1.09)	5.33 (1.11)	**< 0.001**
**Bilirubin, total (umol/L), mean (SD)**	11.47 (7.36)	12.12 (5.45)	12.54 (5.34)	12.78 (4.89)	12.23 (5.85)	**< 0.001**
**Albumin (g/dL), mean (SD)**	4.19 (0.33)	4.21 (0.30)	4.24 (0.32)	4.23 (0.33)	4.22 (0.32)	**0.003**
**ALT (U/L), mean (SD)**	22.99 (58.22)	21.73 (13.14)	22.54 (10.84)	25.17 (12.14)	23.12 (30.81)	**0.038**
**Uric acid (mg/dL), mean (SD)**	4.99 (1.33)	5.51 (1.39)	5.87 (1.40)	6.25 (1.42)	5.65 (1.46)	**< 0.001**
**C-reactive protein(mg/dL), mean (SD)**	0.56 (1.44)	0.51 (1.02)	0.55 (1.02)	0.50 (0.78)	0.53 (1.09)	**0.479**
**Categorical variables**^b^						
**Male, *n* (%)**	156 (12.1)	550 (42.3)	926 (71.5)	1088 (83.8)	2720 (52.4)	**< 0.001**
**Non-Hispanic white, *n* (%)**	808 (62.4)	722 (55.5)	717 (55.3)	688 (53.0)	2935 (56.6)	**< 0.001**
**Coronary heart disease, *n* (%)**	94 (7.3)	129 (9.9)	132 (10.2)	174 (13.4)	529 (10.2)	**0.001**
**Angina/angina pectoris, *n* (%)**	84 (6.5)	112 (8.6)	111 (8.6)	103 (7.9)	410 (7.9)	**0.317**
**Heart attack, *n* (%)**	90 (7.0)	127 (9.8)	145 (11.2)	161 (12.4)	523 (10.1)	**0.001**
**Stroke, *n* (%)**	78 (6.0)	102 (7.8)	112 (8.6)	92 (7.1)	384 (7.4)	**0.171**
**Malignancy, *n* (%)**	254 (19.6)	228 (17.5)	233 (18.0)	215 (16.6)	930 (17.9)	**0.375**
**Smoking, *n* (%)**	173 (28.3)	155 (23.8)	167 (21.4)	133 (15.7)	628 (21.7)	**< 0.001**

Abbreviation: BMI, body mass index; SBP, systolic blood pressure; ALT, alanine aminotransferase;

^a^Values were expressed as mean (standard deviation).

^b^Values in the categorical variables were expressed as number (%).

### Association between HOMA-IR and the MAMC

Multivariable analysis was conducted to reveal the relationship between HOMA-IR and the MAMC in the obesity and non-obesity groups. As shown in Table 4, in the non-obesity group, the MAMC quartiles in the four models had significant positive associations with HOMA-IR (*P* < 0.05). In the obesity group, the MAMC quartiles were only positively associated with HOMA-IR in model 3 and 4; however, the associations between the MAMC quartiles and HOMA-IR in the obesity group were not statistically significant. In addition, among the non-obese group, p for trend was 0.003 in model 1 and p for trends were < 0.001 in model 2, 3, and 4.

**Table 4:** Association between the MAMC and the HOMA-IR in non-obese and obese participants

Models^a^	Quartiles Of MAMC	β^b^ (95% CI)	*P* Value	*P* for Trend	β^b^ (95% CI)	*P* Value	*P* for Trend
		Non-obesity			Obesity		
**Model 1**	Q2 vs Q1 Q3 vs Q1 Q4 vs Q1	0.697 (−0.101, 1.495) 1.050 (0.225, 1.875) 1.269 (0.327, 2.211)	0.087 0.013 0.008	0.003	−0.370 (−3.704, 2.963) 2.729 (−0.548, 6.005) 1.970 (−1.203, 5.142)	0.828 0.102 0.223	0.011
**Model 2**	Q2 vs Q1 Q3 vs Q1 Q4 vs Q1	1.278 (0.434, 2.122) 2.344 (1.316, 3.372) 2.793 (1.616, 3.971)	0.003 < 0.001 < 0.001	< 0.001	−0.488 (−3.840, 2.864) 2.357 (−0.973, 5.687) 1.226 (−2.157, 4.609)	0.775 0.165 0.477	0.123
**Model 3**	Q2 vs Q1 Q3 vs Q1 Q4 vs Q1	0.925 0.164, 1.685) 1.695 (0.764, 2.627) 2.373 (1.309, 3.437)	0.017 < 0.001 < 0.001	< 0.001	0.251 (−2.560, 3.062) 2.366 (−0.424, 5.156) 1.646 (−1.190, 4.482)	0.861 0.096 0.255	0.085
**Model 4**	Q2 vs Q1 Q3 vs Q1 Q4 vs Q1	0.886 (0.124, 1.647) 1.681 (0.749, 2.613) 2.305 (1.237, 3.372)	0.023 < 0.001 < 0.001	< 0.001	0.010 (−2.815, 2.835) 2.150 (−0.656, 4.955) 1.402 (−1.455, 4.260)	0.995 0.133 0.336	0.103

^a^Adjusted covariates:

Model 1 = Unadjusted.

Model 2 = Model 1 + age, gender, race.

Model 3 = Model 2 + serum fasting glucose, serum total cholesterol, serum total bilirubin, serum albumin.

Model 4 = Model 3 + coronary heart disease, angina/angina pectoris, heart attack, stroke, cancer or malignancy.

^b^β coefficients was interpreted as change of the HOMA-IR for each increase in different anthropometric parameters.

## DISCUSSION

We explored the relationship of the MAMC and HOMA-IR in the obesity and non-obesity groups. The most remarkable finding was the significant positive association of the MAMC and HOMA-IR in the non-obesity group. However, the association between the MAMC and HOMA-IR in the obesity group lacked statistical significance. As we have summarized above, the results suggested that the MAMC can be a surrogate indicator for predicting IR in the non-obese population and may be applicable in the future.

The gold standard to evaluate IR is the hyperinsulinemic euglycemic glucose clamping (HEGC) [14]. Nonetheless, the high cost and pump-infusion equipment for obtaining data greatly limit its clinical applicability. HOMA-IR is a less time-consuming, single, portable, less expensive evaluation of IR in clinical practice and research. Matthews *et al.* first described HOMA-IR and utilized it to quantify IR and beta-cell function in 1985 [2]. However, an operational definition of IR by HOMA-IR cannot be set because the HOMA-IR levels may vary due to diverse factors, such as age, gender, ethnicity, and body composition. Evidence has suggested that HOMA-IR is associated with cardiovascular disease (CVD), muscle mass, nonalcoholic fatty liver disease (NAFLD), and several hormones, such as T4, TSH, leptin and adiponectin [15–18]. HOMA-IR is a proxy measurement for IR that has been used in copious surveys [3]. Therefore, the present study adopted HOMA-IR as a measuring tool for IR.

Anthropometric data have been widely applied because they are noninvasive, inexpensive, and convenient to measure. They were closely associated with the psychological status, muscle mass, physical performance, and body composition [19–21]. Lee *et al.* collected anthropometric data and predicted hemoglobin levels in elderly Koreans [22]. They used BMI in combination with weight to improve the predictive power for the hemoglobin level. Past studies on anthropometric data and IR have focused on the WC and HOMA-IR [23, 24]. Bonneau *et al.* explored the role of traditional cardiovascular risk factors as predictors of IR and found that WC was only a predictor of IR in women [23]. A Spanish study indicated that the WC was the best anthropometric parameter correlated with IR in children [24]. Other studies used the mid-arm muscle to yield valuable data [25, 26]. The mid-arm muscle area (MAMA) can be a better predictor of mortality than BMI in chronic obstructive pulmonary disease (COPD). The MAMA also can estimate the prognostic influence of muscle mass depletion [25]. The MAMC and TS thickness may assist in the biochemistry analysis of undernutrition status [26]. Among maintenance hemodialysis (MHD) patients, a higher MAMC represented larger lean body mass and better mental health and survival rate [12]. Another study conducted in the United Kingdom suggested that high WC and low MAMC predisposed elderly men to higher mortality and we should employ these two measures in assessing the body composition and mortality in the elderly [27].

Mediated by insulin, skeletal muscle is a main source of glucose metabolism and can profoundly influence IR. There are type I and II muscle fibers, which can be distinguished by their enzymatic capacity and myosin heavy-chain isoforms [28]. Type I fibers are mitochondria-rich with high oxidative capacity, whereas type II fibers have fewer mitochondria and a lower oxidative capacity [29, 30]. Muscle fibers are influenced by genes and obesity [31–34]. Fat diet-related obesity may induce the transition of muscle fibers from type I to type II [35]. Another widely known risk factor for IR is obesity, defined by BMI ≥ 30.0 kg/m^2^ according to the Centers for Disease Control and Prevention (CDC) [36, 37]. In a Korean study, sarcopenia and obesity exerted a combined effect on IR [8].

The study conducted by Atkins *et al.* demonstrated a salient association between HOMA-IR and the MAMC [13]. The participants were divided into MAMC quartiles and the MAMC was positively associated with the BMI. The results initially showed that the HOMA-IR increased in the higher MAMC quartiles. After adjusting for the BMI, the HOMA-IR level was related to increased odds of low MAMC. The direction of the association between HOMA-IR and the MAMC changed after adjustment because there was a strong relationship between obesity and IR. Therefore, we re-evaluated the relationship between HOMA-IR and the MAMC after dividing the participants into obesity and non-obesity groups. In our study, the MAMC was positively associated with HOMA-IR in the non-obesity group, which may be explained by the positive association between the MAMC and BMI, as observed in previous studies. While the MAMC and BMI increased, the levels of HOMA-IR also increased. By contrast, the association between the MAMC and HOMA-IR was nonsignificant in the obesity group.

We reason that the MAMC can be a surrogate for HOMA-IR in the non-obese group. However, we cannot utilize the MAMC to predict HOMA-IR in obese individuals. In obese people, there is a higher lipid level within the skeletal muscle [38]. Additionally, high fat diet-induced obesity galvanizes the transition of type I muscle fibers to type II, which are less responsive to insulin [35]. These observations may be associated with diminished muscle insulin sensitivity [39] and increased IR in the obese population. In articles of healthy participants, it has been claimed that “at BMI > 30 kg/m^2^, nearly all subjects had low insulin sensitivity” [40]. We surmise that the baseline IR is higher in obese people; therefore, when the MAMC is increased, the change in IR is not observable.

There are a few limitations in the present study. First, the cross-sectional nature of our survey prevented us from drawing causal inferences among the observed variables. In addition, an observational investigation can assess relationships, but it does not provide information about causations. Second, the anthropometric data and blood sampling were obtained at one time point without long-term follow up. Therefore, we could not observe continuous changes. Third, we needed to consider reporting bias because we used NHANES database, which contained a myriad of self-reported variables. Moreover, the NHANES program was designed for the people in the United States, and the study results are not suitable for extrapolation to a larger population. Fourth, insulin sensitivity is related to percentage of body adipose tissue, even within relatively normal ranges of BMI [41]. Since NHANES 1999–2006 failed to include fat mass as one of the variates, the survey cannot adjust for fat mass. Adipose tissue within the skeletal muscle limits the ability of MAMC to accurately estimate muscle mass [42]. In this respect, it is salient to highlight that no statistical significance between MAMC with HOMA-IR in the obese group observed in our study may be partially due to the inability to assess the excessive amount of fat accumulated outside and inside the muscle. Finally, there were unmeasurable confounding factors that cannot be excluded even though we adjusted for the residual confounders.

In summary, the MAMC had a significant positive association with HOMA-IR in the non-obesity group, but their relationship was not statistically significant in the obesity group. Therefore, the MAMC may be used as a surrogate indicator for predicting HOMA-IR in non-obese people. Our study findings are provocative. Considering that anthropometric data are accessible and easy to measure for frail or ill people, future studies should replicate our results and further explore the associations between anthropometric data and HOMA-IR to develop early detection methods for IR.

## MATERIALS AND METHODS

### Ethics statement

This investigation passed the following formal review procedures: the Institutional Review Board (IRB) review in the United States and an online protocol management and review system. Appropriate steps in the research were followed in accordance with the IRB-approved protocol. The rights and welfare of the participants were protected by obtaining consent for participation.

### Data source and participants

The data were obtained from the 1999–2006 NHANES, which was conducted by the CDC. The study was performed in accordance with the Declaration of Helsinki. The goal of NHANES survey is to produce valuable health statistics for the Nation. NHANES began in the early 1960s and has focused on health and nutritional measurements since 1999. The samples included were used to represent all ages of the population in the United States. However, because of the aging population, which may soon become the most important, NHANES over-sampled persons aged 60 or older. All participants participated in a physician interview and body measurements. Health interviews were performed in respondents’ homes and measurements were conducted in specially designed mobile centers. NHANES is an ongoing program based on a multi-stage sample plan. More details of the sampling, measurement, and laboratory data are available on the CDC website. The participants were divided into two groups based on their BMI, the obese (BMI ≥ 30 kg/m^2^) group and non-obese (19 ≤ BMI < 30 kg/m^2^) group. Each group was then divided into four subgroups based on their MAMC levels.

### Measurement: insulin resistance

Normally, the insulin level increases as glucose is released from carbohydrates to the bloodstream. IR is a pathological condition with impaired ability of insulin to stimulate glucose utilization, and it is a major cause of non-insulin-dependent diabetes mellitus (NIDDM). The gold standard to measure insulin sensitivity is HEGC [14]. With the incorporation of radioactive-labeled glucose during euglycemic clamps, HEGC can measure glucose metabolism in individual organs. Nonetheless, HEGC is labor intensive and time-consuming, and it requires experienced operators. Therefore, we adopted HOMA-IR, a simple surrogate index for IR that is derived from blood insulin and glucose under fasting conditions (steady state) or after an oral glucose load (dynamic) for measuring IR. HOMA-IR is calculated by the following equation: HOMA-IR = [fasting serum insulin (mU/L) · fasting plasma glucose (mmol/L)]/22.5 [2]. According to the World Health Organization (WHO), IR is usually defined as a value greater than the 75th percentile value for non-diabetic subjects. Radikova *et al.* used the 75th percentile value as the cut-off point to define IR corresponded with a HOMA-IR of 2.29 among Caucasian rural population [43]. We used the HOMA-IR value 2.29 to judge the existence of IR in our study because a great majority of our participants were Non-Hispanic whites.

### Measurement: anthropometric parameters

The definition of BMI is a person’s weight (kilograms) divided by the square of the height (meters), and the unit of BMI is kg/m^2^. The operator stood behind the subject to locate the middle of the upper arm with a mark (+) and the subject stood upright with his or her shoulder relaxed and right arm pendant. The operator placed measuring tape around the mark (+) perpendicular to the axis of the right arm. While obtaining the mid-upper arm circumference (MAC), the tape was close to the skin without overt tightness. The staff recorded the MAC value to the nearest 0.1 cm. To measure the TS, the operator grasped a fold of skin and adipose tissue above the MAC mark (+) and held the skin fold parallel to the long axis of the arm. Then, the jaws of the caliper were placed over the mark point. The staff recorded the TS value to the nearest 0.1 mm with fingers continuously holding the skinfold. We calculate the MAMC (cm) as MAC (cm) −0.3142 × TS (mm). To measure the WC, subjects stood first and the operator palpated and marked the upper lateral border of the right ilium. The measuring tape was placed at the mark site and the operator measured around the trunk in a horizontal plane. The staff measured the WC to the nearest 0.1 cm when the subject was in the minimal respiration phase and the tape was snug without compressing the skin. For the TC measurement, the subject was in the standing posture with most of the weight on the left leg and then protruded the right leg with the knee slightly flexed and soles flat on the floor. The operator marked the mid-thigh and positioned the tape around the mark to retrieve the TC value to the nearest 0.1 cm. The measuring tape was rested on the skin surface without tightness. The MCC was measured while the subject was sitting. The operator placed the tape around the calf and moved the tape perpendicular to the long axis of the calf to locate the maximal circumference. The MCC was recorded to the nearest 0.1 cm. The operator measured the SS with the subject standing erect and shoulder relaxed and then grasped a skinfold above and medial to the inferior scapular angle. The tips of the caliper jaws were placed perpendicular to the length of the fold, and the operator measured the skinfold to the nearest 0.1 mm while the fingers continued to hold the skin.

### Measurement: outcomes

Independent of obesity, sarcopenia is associated with dysregulation of glucose metabolism [9]. The prevalence of overweight and obesity has been increasing, and these are risk factors for IR [44, 45]. We sought new methods that can assist in early detection of IR.

This investigation explores the relationship between HOMA-IR and anthropometry data after dividing the participants into obese and non-obese groups. The anthropometry data included in this study are as follows: WC, MCC, TC, TS, SS, and MAMC, and these data were collected form the NHANES III database. A previous study illustrated that the MAMC and HOMA-IR were positively associated; however, the study results were influenced by BMI. Therefore, we separated the participants into obese and non-obese groups and evaluated the relationships between the two groups and the HOMA-IR values. HOMA-IR in this investigation was calculated from the blood glucose and insulin concentration of the participants. All study procedures were performed with informed consent and had been approved by the IRB.

### Measurement: covariates

Gender, age, race, smoking status and past medical history were self-reported covariates. Past medical history was diagnosed by a doctor, including coronary heart disease, angina/angina pectoris, heart attack, cancer, and stroke.

The HNAHES database obtained metabolic variables from individuals’ blood samples. To analyze the plasma glucose, venipuncture was performed after fasting for 6 hours. The Cobas Mira Chemistry System (Roche Diagnostic Systems, Indianapolis, IN, USA) was adopted and the hexokinase enzymatic method was used to analyze the plasma sugar. Insulin radioimmunoassay (RIA), a double-antibody batch method, was used to measure insulin level.

To measure the serum total cholesterol, the Hitachi 704 Analyzer (Roche Diagnostics, Indianapolis, IN, USA) was used and the serum CRP level was measured by latex-enhanced nephelometry (Behring Nephelometer II Analyzer System; Behring Diagnostics Inc., Somerville, NJ, USA). For other biochemical profiles, such as the serum albumin, serum UA and ALT, the Beckman Synchron LX20 instrument was used. The CDC approved the standard protocols of assessing the data during the data collection period.

### Statistical analysis

We used Predictive Analytics Suite Workstation Statistics (SPSS; SPSS Inc., Chicago, IL, USA) for data management. SPSS is a univeral software that assisted with our statistical analysis. Considering progressive modification, multivariable logistic regression analysis was used to observe the association between the anthropometry data (WC, MCC, TC, TS, SS, and MAMC) and HOMA-IR. We performed covariate adjustments with the model-adjusted method. No variables were adjusted in Model 1. Model 2 consisted of Model 1 adjusted by age, gender, and ethnicity. Model 3 consisted of Model 2 adjusted by glucose, total cholesterol, total bilirubin and albumin. Model 4 consisted of Model 3 adjusted by angina/angina pectoris, heart attack, stroke and malignancy or cancer. We analyzed the categorical variables by the Chi-square test, while we analyzed the continuous variables by ANOVA. Among all variables, the categorical variables were expressed as numbers and percentages, and the continuous variables were expressed as the means and standard deviations (SD). The MAMC values of the study participants were examined in quartiles. A *P value* that was more than 0.05 indicated a lack of a significant difference. After dividing the participants into the obesity and non-obesity groups, the association of the MAMC and HOMA-IR in the two groups was analyzed by ANOVA using an R function (R ANOVA). To evaluate the association of Q2/Q1, Q3/Q1, and Q4/Q1, trend analysis were used in both groups and all the designed models. According to the multivariable Cox proportional hazard ratio models, we classified the subjects into the unadjusted model (Model 1) and adjusted models (Model 2–4) and then evaluated the association between the MAMC and HOMA-IR in the obesity and non-obesity groups.
